# Using Camera Trapping to Assess the Status of the Mammalian Community in the Mafou Fully Protected Area, Upper Niger National Park (Guinea)

**DOI:** 10.3390/ani16142151

**Published:** 2026-07-11

**Authors:** Mahutin Bruno Ganvoedjre, Estelle Raballand, Dylan Deffaux, Marius Kabongo, Siaka Oularé, Serge Alexis Kamgang, Cédric Vermeulen

**Affiliations:** 1 Regional Post-Graduate Training School on Integrated Management of Tropical Forests and Territories (ERAIFT/UNESCO), University of Kinshasa, Kinshasa P.O. Box 15373, Democratic Republic of the Congo; sergekamgang@yahoo.fr (S.A.K.); cvermeulen@uliege.be (C.V.); 2 Chimpanzee Conservation Center (CCC), Faranah P.O. Box 36, Guinea; founder@chimpanzeeconservationcenter.org (E.R.); dylan.deffaux@gmail.com (D.D.); kabongomarius@gmail.com (M.K.); 3 Upper Niger National Park (UNNP), Guinean Office of National Parks and Wildlife Reserves (OGPNRF), Conakry P.O. Box 634, Guinea; siaka.oulare@gmail.com; 4 Garoua Wildlife School (EFG), Garoua P.O. Box 271, Cameroon; 5 Forest Is Life, Gembloux Agro-Bio Tech, University of Liège, 5030 Gembloux, Belgium

**Keywords:** camera trap, Upper Niger National Park, protected area, terrestrial mammals, threatened species, mammal assemblage, occupancy

## Abstract

The Upper Niger National Park (UNNP) is the oldest park and one of the most promising conservation areas in Guinea; yet, its mammalian fauna remains poorly documented. Camera trapping has become an essential tool for revealing the diversity and assemblage structure of such communities. This study employed camera traps to improve knowledge about terrestrial and semi-terrestrial mammals with body mass > 0.5 kg (Sciuridae and heavier) in the Mafou Fully Protected Area (Mafou FPA), the principal core zone of the UNNP. The survey was conducted during the dry season, from January to May 2025. Sampling targeted animal trails within forest habitats in the Mafou FPA and involved the deployment of 53 camera traps with an average inter-trap distance of 2 km. Across 4239 camera-days of sampling effort, we collected 10,334 usable images and videos, yielding 2634 independent detection events. Thirty taxa across 15 families and five orders, including six species of high conservation concern according to the IUCN Red List (version 2025-1) were recorded. The mammal assemblage consists of species from the three known trophic levels (prey, mesopredators, and apex predators), with predominance of medium-sized prey species. Our results demonstrate a remarkable richness in frugivorous seed-dispersing species, ecosystem-engineering species, and the presence of a megaherbivore, which all contribute to its ecological dynamics. Despite a notable human activity index of 0.42, occupancy models revealed broad spatial distribution of medium-sized mammals within this protected area. The occupancy patterns demonstrated that occurrence of some species might be sensitive to human disturbances in the area. These findings are an important contribution to Guinean and West African biodiversity assessments. They highlight the critical conservation value of the UNNP within the subregion.

## Simple Summary

In this study, we used data from 53 camera traps set up between January and May 2025, to document the mammalian community of the Mafou Fully Protected Area in Upper Niger National Park (Guinea). From 10,334 images across 4239 camera-days, we recorded 30 taxa across 15 families and five orders, including six threatened species, such as *Pan troglodytes verus*, *Smutsia gigantea*, *Colobus polykomos*, *Hippopotamus amphibius*, *Panthera pardus* and *Phataginus tricuspis*. The study area shows wide spatial distribution of medium-sized mammals and a mammal assemblage dominated by herbivores. Despite human activity and poaching risks, these findings underscore the park’s critical conservation value for regional biodiversity. This study establishes a crucial biodiversity baseline for species monitoring in the Mafou Fully Protected Area.

## 1. Introduction

Biodiversity loss represents one of the most profound and irreversible anthropogenic changes of the twenty-first century. In response to this global challenge, protected areas constitute a cornerstone for safeguarding biodiversity [1], providing essential ecological functions and ecosystem services for human well-being [2]. However, multiple pressures including demographic growth, shifting land-use patterns, and the degradation of natural habitats, intensify threats to conservation areas, particularly in West Africa. Rural populations have increasingly expanded toward the peripheries of protected areas [3], thereby accelerating land-use conflicts and ecological degradation.

The Upper Niger National Park (UNNP) of Guinea is no exception. As one of the country’s most biologically important protected areas [4,5], it hosts numerous species that do not occur in other national conservation units. Yet, its mammal populations have experienced marked declines in recent decades, with reductions in both abundance and species diversity [5]. Major drivers include deforestation, poaching, and artisanal fishing even within the Mafou Fully Protected Area (FPA), the ecological core of the UNNP [6,7,8].

Since 2005, no multi-species wildlife study has been conducted to evaluate the status and trends of the mammalian community in this protected area. This absence of long-term monitoring reflects broader regional limitations, as many of Guinea’s protected areas lack adequate data and resources for biodiversity assessment [9,10], whereas regularly estimating species richness and abundance is crucial to documenting species declines, understanding ecological and anthropogenic factors impacting wildlife communities and assessing the positive outcomes of wildlife management and conservation activities [11,12]. Given these constraints, camera trapping represents a cost-effective and powerful tool for wildlife monitoring, allowing continuous, non-invasive data collection across landscapes [13,14,15]. This method is especially relevant in West African contexts where logistical and financial limitations impede traditional survey techniques [16].

Although camera trapping has expanded substantially across the continent, its use in Guinea remains extremely limited [17,18]. In particular, no camera trap-based multi-species research had previously been conducted in the UNNP. Considering the ongoing anthropogenic pressures and the ecological importance of the park, there is an urgent need to update information on its mammalian fauna to inform conservation strategies and guide management priorities [19]. This study aimed to fill this knowledge gap.

It is implemented as part of the “Camera Trap Biomonitoring Project” of the Chimpanzee Conservation Center (CCC), first launched in 2018 with the main goal of the wild Western Chimpanzee monitoring in the Mafou FPA of the UNNP. Specifically, we sought to: (i) quantify the presence of terrestrial and semi-terrestrial mammals in the Mafou FPA; (ii) assess its mammal assemblage structure; and (iii) model occupancy patterns to better understand species distribution and the influence of human-related disturbances.

## 2. Materials and Methods

### 2.1. Study Area

**Figure 1:**
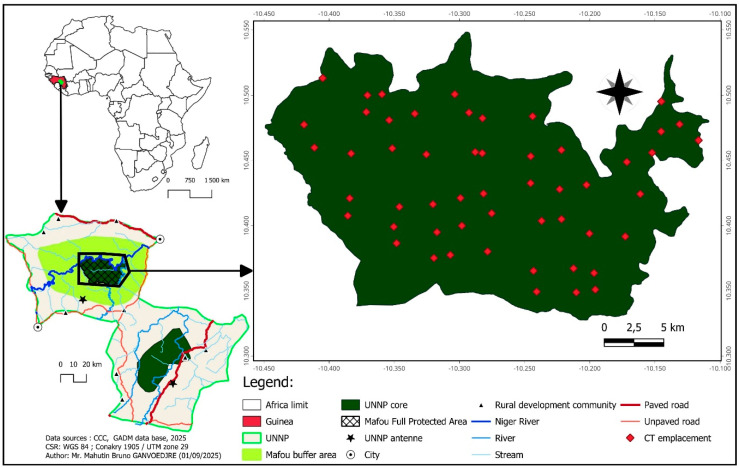
Location of the study area in Guinea, and network of camera traps installed in Mafou fully protected area of the Upper Niger National Park.

The Upper Niger National Park (UNNP), established by Decree D/97/011/PRG/SGG on 28 January 1997, is located in the natural region of Upper Guinea, which lies between 8 and 11°37′ N and 8°45′–12°35′ W. The park covers approximately 12,470 km^2^ and spans the prefectures of Kouroussa, Dabola, Faranah, Kankan, and Kissidougou (Figure 1). It is composed of two management sectors: Mafou sector (6470 km^2^) and Kouya sector (6000 km^2^), each with a central core designated as a Fully Protected Area (FPA). This study focuses on the Mafou FPA, corresponding to the Mafou Classified Forest (554 km^2^), surrounded by an extensive buffer zone [20].

Designated as a Ramsar site in 1992 due to the ecological importance of the Niger River, the UNNP was recognized as a UNESCO Biosphere Reserve in 2002. The local population consists predominantly of Malinké communities, with an average density of 7.3 inhabitants/km^2^ in the Mafou sector. The climate is Sudano-Guinean, characterized by a pronounced dry season (November–May), and the rainy season from June to October. According to NASA’s data [21], mean temperatures are around 26°, and average annual rainfall is approximately 1500 mm.

### 2.2. Data Collection

Camera trapping was carried out during the dry season, from 23 January to 19 May 2025, to ensure adequate sampling effort [13,22,23]. We used the point-transect method adapted for camera trapping [24], with all units operating continuously, day and night. A sampling framework targeting animal trails has been designed following a 3 km × 3 km grid [25], using QGIS version 3.34.4 software. In total, 53 camera traps were deployed, one per grid cell, to achieve full coverage of the study area. These cameras included 30 HunterCam PR1000 (Shenzhen Hunting Tech Co., Ltd., Shenzhen, China), 15 GardePro A3S (GardePro, Hong Kong, China), 5 Solognac BG500 (Decathlon, Incheon, Republic of Korea), and 3 ENKEEO PH730S (ENKEEO, Shenzhen, China).

Within each grid, we systematically searched for field indicators such as active wildlife trails or junctions, visible chimpanzee nests, fresh footprints, and especially water sources in forested habitats, notably the gallery forests, to set camera traps [26,27]. Once a site was selected, cameras were mounted on trees or shrubs at heights between 0.40 and 0.70 m, depending on local topography. Orientation was north–south, with slight east–west adjustments when needed to ensure that a sufficient length of the target trail fell within the detection zone [28]. This set-up approach produced an average inter-camera distance of roughly 2 km (Figure 1).

A data collection database was built in SMART version 7.5.9 and linked to the SMART Mobile APK465 application [29], enabling systematic recording of installation and monitoring information throughout the campaign. Recorded variables included camera ID, habitat type, orientation, wildlife indicators, and activation hour. For each detection event, the cameras captured two consecutive photographs with a 5 s trigger delay, followed by a one-minute video. All units were checked every two weeks to replace batteries and SD cards and to identify and resolve any malfunctions.

### 2.3. Species Identification and Encoding

To ensure reliable species identification and reduce potential bias, we applied a two-stage manual identification process, drawing on the Guide to the Identification of African Mammals [30] and the Guide to the Identification of Morphologically Similar or Rare Mammal Species in Central Africa Using Camera Traps [31]. The first stage consisted of pre-identification, during which all images and videos were reviewed using Windows Media Player. False positives, such as triggers caused by wind, insects, or bushfires, were removed, and all files containing animals or other relevant information (e.g., human presence) were renamed to reflect their content. Final identification and simultaneous data coding were then performed in Timelapse version 2.3.0.0 software, a widely used program for processing camera-trap datasets [32,33]. At this stage, only terrestrial and semi-terrestrial mammals were considered. When species could not be confidently distinguished, they were grouped into three morphological complexes: *Genetta pardina* and *Genetta maculata* were assigned to “*Genetta* spp.”; indistinguishable mongoose species were grouped under “Herpestidae”; and squirrel species (*Heliosciurus rufobrachium*, *Heliosciurus gambianus*, *Funisciurus pyrrhopus*, *Protoxerus stangeri*, and *Epixerus ebii*) were categorized as “Sciuridae.” Sequences for which no identification was possible were classified as “Not identified.”

### 2.4. Data Analysis

To analyze the dataset generated by this study, we first used the R Shiny application “EuRÊCAM!”, developed within the FauneFac and CAAPP-Faune projects and hosted by Forest is Life, Gembloux Agro-Bio Tech, University of Liège (https://www.gembloux.ulg.ac.be/faunefac/, accessed on 29 August 2025). This tool facilitates the processing and exploration of camera-trap data [33]. Using EuRÊCAM!, we calculated sampling effort and species richness, determined rarefaction curves, and we estimated detection rates for each recorded species. To reduce the risk of overestimation, we considered detections to be independent only when separated by at least 30 min for the same species [34]. When multiple detections (more than 2 captures) of the same species occurred within this 30 min window, only the event with the highest number of individuals was retained.

In the second phase, we assessed mammal assemblage structure on the basis of the mean adult weight of the species and their trophic guild. We defined the small mammal category based on the criteria of Degen [35] and Merritt [36]: body mass < 5 kg. Medium-sized mammals are considered to weigh between 5 kg and 100 kg, while large mammals are defined as having an average adult weight of more than 100 kg.

Finally, we estimated and modeled occupancy for the 19 species and species groups that registered at least 10 independent detections [37], following the approach described by MacKenzie et al. [38]. This approach requires assumptions, including the area’s closure during the survey period, as argued by Rota et al. [39], Kéry et al. [40], and Bailey et al. [41], independence among sampling sites, and appropriate survey duration. Analyses were conducted in Program PRESENCE version 2.15.18 [42], using a single-season occupancy model [43]. For each species, we constructed a detection history based on five 8-day sampling occasions according to the recommendations of MacKenzie & Royle [44] and Ancrenaz et al. [45]. In this matter, missing data, due to camera trap malfunctions, were coded with a point (.).

Occupancy modeling incorporated two site-level covariates representing anthropogenic pressures. The first, Habdist, is the distance from each camera station to the village of Sérékoroba, the nearest human settlement, calculated using QGIS. Despite the CCC sanctuary and its release-site camp being located at the margins of the Mafou FPA, they were not considered sources of disturbance affecting species occupancy. The second covariate, Hact, corresponds to the number of illegal-activity indicators (georeferenced) recorded in each grid cell, derived from the CCC database. These indicators collected during park guards’ patrols organized by the CCC throughout the Mafou FPA between January 2024 and June 2025 include primarily spent cartridges, along with evidence of bush fires, agricultural clearing, honey extraction, and wood cutting. The survey effort for this stage of data collection was 12,414 km traveled during 214 patrols. For each grid cell, all indices were summed and assigned to the associated camera trap. We weighted all illegal activities equally in order to avoid any potential biases related to their categorization during data collection.

Because the survey was carried out entirely during the dry season under similar weather conditions, we assumed constant detection probability across the study area, even though we were aware that detection could be influenced by certain covariates, such as sampling effort and others. For each species, we evaluated all combinations of covariates for occupancy and detection: *ψ*(.)*p*(.); *ψ*(Hact)*p*(.); *ψ*(Habdist)*p*(.); and *ψ*(Hact + Habdist)*p*(.).

Models were ranked using Akaike’s Information Criterion (AIC), with ΔAIC < 2 indicating suitable models [46]; the best-fitting models were those with ΔAIC = 0.00. For species whose occupancy probability were influenced by a covariate, we examined the corresponding beta coefficients to interpret the direction and strength of these effects [47]. Some graphs were created in RStudio version 4.5.2 using the ggplot2 package version 4.0.3.

## 3. Results

### 3.1. Sampling Effort

At the end of the survey, 40 camera traps were functioning normally, while 13 experienced temporary malfunctions. From a total of 10,334 useful images and videos (representing observations of mammals or humans), we accumulated a sampling effort of 4239 camera-days, recording 2634 independent events. This effort exceeds the minimum threshold of 1000 camera-days recommended to detect rare species [25,26].

### 3.2. Specific Richness and Detection Rates

A total of 30 species and species groups of terrestrial and semi-terrestrial mammals were recorded in the Mafou FPA, distributed across 5 orders and 15 families (Table 1).

These findings confirm the presence of six species of high conservation concern, as classified by the IUCN Red List (version 2025-1). *Hippopotamus amphibius* and *Panthera pardus* are listed as Vulnerable, *Phataginus tricuspis*, *Smutsia gigantea*, and *Colobus polykomos* as Endangered, and *Pan troglodytes verus* as Critically Endangered. Notably, the study area hosts a remarkable blend of forest and savanna mammals, underscoring its ecological uniqueness.

**Figure 2:**
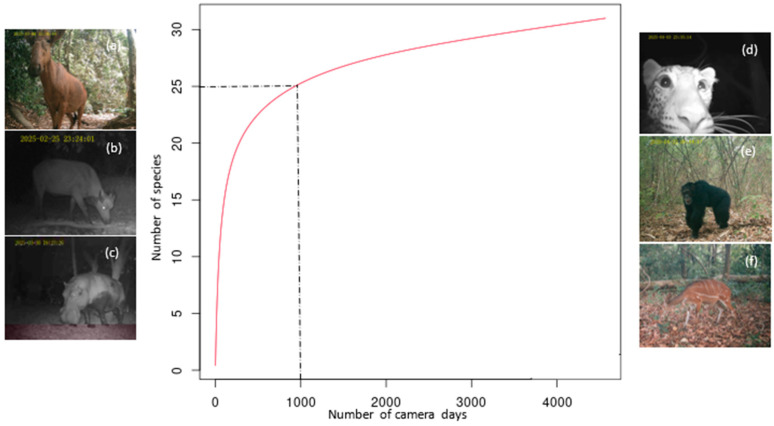
Rarefaction curve showing the number of detected mammal species as a function of the cumulated number of camera-days, illustrated by some mammals detected during this study in the Mafou FPA, 2025. (**a**) *Alcelaphus buselaphus major*; (**b**) *Syncerus caffer nanus*; (**c**) *Hippopotamus amphibius*; (**d**) *Panthera pardus*; (**e**) *Pan troglodytes verus*; (**f**) *Tragelaphus scriptus*.

The rarity curve (Figure 2), which illustrates the cumulative number of species detected relative to sampling effort, indicates that 83.33% (25 species) of all recorded species were documented within the first 1000 camera-days. Given that more than 4000 cumulative camera-days were deployed, the curve’s asymptote suggests that the inventory is close to complete; detecting additional species would likely require substantially greater effort.

Detection rates show that *Pan troglodytes verus* is the most frequently recorded species, with the highest Relative Abundance Indices (RAI = 13.353). In contrast, *Leptailurus serval*, *Lupulella adustus*, and *Smutsia gigantea* were rarely detected, each with only a single record and RAI values of 0.024. Among other large- and medium-sized mammals, *Cephalophus maxwelli* (RAI = 7.007) and *Tragelaphus scriptus* (RAI = 5.733) are relatively common, followed by *Potamochoerus porcus* (4.388), *Cercopithecus sabaeus* (3.987), and *Cephalophus silvicultor* (1.911). They are followed by *Cephalophus rufilatus* (1.605), *Phacochoerus africanus* (1.298), *Papio papio* (1.251), *and Erythrocebus patas* (0.732). Among smaller mammals, *Cricetomys gambianus* (6.252) and members of the family Herpestidae (4.176) show the highest detection rates.

Of the six IUCN-listed threatened species, only *Pan troglodytes verus* shows a relatively high detection rate. The remaining five species exhibit low RAIs, estimated at 0.118 for *Hippopotamus amphibius*, 0.189 for *Phataginus tricuspis*, 0.166 for *Panthera pardus*, 0.024 for *Smutsia gigantea*, and 0.095 for *Colobus polykomos*.

### 3.3. Mammal Assemblage Structure

**Figure 3:**
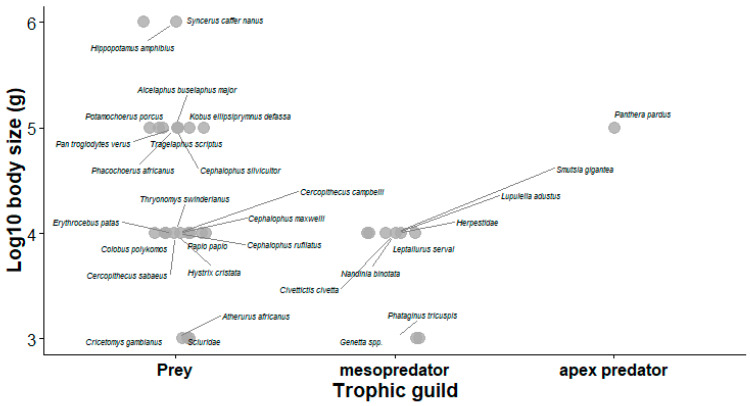
Distribution of mammal species in the Mafou Fully Protected Area, on the basis of body size.

The mammal assemblage in the Mafou FPA comprises species from the three known trophic guilds: primary consumers/prey, secondary consumers/mesopredators, and tertiary consumers/apex predators (Figure 3). Prey are by far the most dominant group, accounting for approximately 70.97% of the community species, followed by mesopredators which represent 25.8%, while the apex predator *Panthera pardus* stands alone (3.23%) at the top of the trophic guild.

Considering body weight criterion, this assemblage is primarily dominated by medium-sized mammals, with large mammals representing a minority of the species. The number of medium-sized mammal species recorded accounts for approximately 54.84% of the total number of species recorded in this community, compared with 29.03% and 16.13% for small mammals and large mammals, respectively.

The dietary criterion allows for the identification of four main groups: insectivores (6.45%), omnivores and carnivores each in a proportion of 22.58%, herbivores (48.39%), with the notable presence of *Hippopotamus amphibius*, a megaherbivore and ecosystem-engineer (Table 1). In general, medium-sized prey constitute the dominant group of the assemblage. We should also note the strong presence of frugivorous seed-dispersing species, which represent more than 45% of the total number of species recorded.

### 3.4. Occupancy Estimation and Modeling

Of the 30 species and species groups recorded, only 19 accumulated at least 10 independent detections and were therefore included in the occupancy analyses presented in Table 2. For each of these species, the best-fitting model was selected from the four candidate models tested (see Supplementary Materials). The results indicate that *Tragelaphus scriptus* and *Potamochoerus porcus* are the most widely distributed species across the study area, with occupancy estimates of 0.84 (±0.06) and 0.80 (±0.07), respectively. *Erythrocebus patas*, *Civettictis civetta*, *Cercopithecus sabaeus*, and *Cephalophus maxwelli* also exhibit broad spatial distributions, with occupancy values of 0.78 (±0.32), 0.74 (±0.67), 0.73 (±0.08), and 0.73, respectively. They are followed by *Genetta* spp., with *ψ* = 0.63 (±0.10), and *Phacochoerus africanus*, whose occupancy is estimated at 0.62 (±0.12).

Approximately 68% of the 19 species with at least 10 independent detections occupy more than half of the study area. This pattern indicates that mammals are, overall, widely distributed across the Mafou FPA. *Pan troglodytes verus* and *Cephalophus maxwelli* show the highest detection probabilities, with estimated *p* values of 0.56 (±0.04) and 0.52 (±0.04), respectively. In contrast, *Civettictis civetta*, *Erythrocebus patas*, *Syncerus caffer nanus*, and the *Sciuridae* group exhibit the lowest detection probabilities, estimated at 0.05 (±0.05), 0.11 (±0.05), 0.13 (±0.07), and 0.14 (±0.05).

The occupancy models indicate that covariates Hact (illegal activities), and Habdist (distance from human dwellings) influence the spatial occupancy of 6 of the 19 species. *Cephalophus maxwelli*, *Cephalophus silvicultor*, and *Syncerus caffer nanus* have possible association with Hact, whereas the occupancy of *Cephalophus rufilatus*, *Papio papio*, and *Pan troglodytes verus* might be affected by distance from the village of Sérékoroba, the nearest settlement to the Mafou FPA.

Beta coefficients (Table 3) show that illegal activities have a negative effect on the occupancy of *Cephalophus maxwelli* and of *Cephalophus silvicultor*, while *Syncerus caffer nanus* is more frequently detected in areas where illegal activities were more commonly recorded. *For Cephalophus rufilatus*, *Papio papio*, and *Pan troglodytes verus*, increasing distance from Sérékoroba corresponds to greater occupancy. This suggests that these three species are absent or rare near the village, with the effect being strongest for *Papio papio* and even more pronounced for *Cephalophus rufilatus*.

## 4. Discussion

The results of this study cannot be directly compared with those of Touré et al. [58] and Camara [5], as the data collection methods used in those studies differed substantially. Nevertheless, while our survey documented 30 species and species groups comprising 10 Artiodactyla, seven Carnivora, two Pholidota, six Primates, and five Rodentia, Camara [5] recorded only 18 mammal species in the same area (10 Artiodactyla, four Carnivora, three Primates, and a single rodent species) with direct observation using the linear transect method. This contrast highlights the superior effectiveness of camera traps for mammal inventories [13]. The trail-oriented placement strategy used here could introduce detection bias toward trail-using species, and, as a result, would affect the occupancy estimate. Species that use trails, forest habitats or chimpanzee-associated areas may be overrepresented, whereas savanna-associated, arboreal or less trail-using species may be under-detected. However, it performs comparably to systematic camera orientation approaches in terms of species richness and overall community composition, while having only a limited influence on detection rates [59].

Although our survey confirms the presence of the species detected, it does not exclude the possibility that some species went undetected [45]. For instance, *Kobus kob kob*, previously reported from the area by Camara [5], was not captured by our cameras, yet a group was directly observed in a grassland within the FPA during fieldwork, confirming its continued presence. Its non-detection likely stems from our sampling focus on forest habitats, whereas *Kobus kob kob* is typically associated with savanna grasslands [60]. This same habitat bias likely explains the absence of *Ourebia ourebi*, a species both scarce in the UNNP [5] and characteristic of savanna environments. Similarly, under-detection for other grassland and/or savanna-associated species, such as *Leptailurus serval*, may reflect a mismatch between survey design and habitat preference. Despite this, the UNNP retains conservation value due to its unique combination of forest and savanna fauna. This specificity, which reveals the uniqueness of the UNNP in the protected areas network of Guinea, is justified by the fact that it covers an ecological transition zone between the forest ecosystems of the South and the savannas of the North, thus resulting in a high biological diversity as well as the presence of species that are on the margin of their distribution area [61]. Moreover, our survey clarified the presence of *Syncerus caffer nanus* in the park, information absent from previous studies, which listed only *Syncerus caffer* without specifying the subspecies.

Regarding species abundance, several authors [62,63] have emphasized that Relative Abundance Indices (RAIs) do not account for variation or imperfections in detectability. Consistent with Ancrenaz et al. [45], they caution against using RAIs as true abundance measures, suggesting instead that they be treated as indicators of capture success. The fundamental problem with this method is that, in order to make valid comparisons between species, space, and time, it is necessary to assume that the detectability of species is constant across the aforementioned dimensions [63]. A more robust alternative is to use occupancy as a proxy for abundance [62]. Occupancy modeling is especially suitable for species that cannot be individually identified, as it assesses space use while explicitly incorporating imperfect detection [45]. The inclusion of covariates further strengthens this framework, making it a statistically rigorous tool [64] with strong parallels to capture–recapture methods [62]. Regarding the assumptions required for estimating and modeling occupancy, the Mafou FPA is first largely bounded by major waterways: the Niger River to the northwest and north, and the Mafou River to the east and southeast. Second, the majority of the species studied are territorial, and the chosen sampling period (five 8-day periods) is relatively short. Furthermore, in the context of the Upper Niger National Park, there are no known species that migrate seasonally to other protected areas. These limit contact with the surrounding area, and consequently limits migration possibilities. However, this does not completely rule out the possibility of contact with the outside. Nevertheless, several authors such as Bruce et al. [65] and Fotsing & Kamkeng [66], have assessed the occupancy of mammal communities in the Dja Wildlife Reserve and the Mpem and Djim National Park in Cameroon, respectively, under similar conditions. Otherwise, our sampling, with an average distance of approximately 2 km between camera traps, is based on the principle that a species detected by a given camera trap is not detected by another, thus assuming independence among sites (cameras). However, this hypothesis regarding the movement of species must be taken into account when exploitation occupancy estimates for species that are likely to travel long distances, such as *Pan troglodytes verus*.

**Figure 4:**
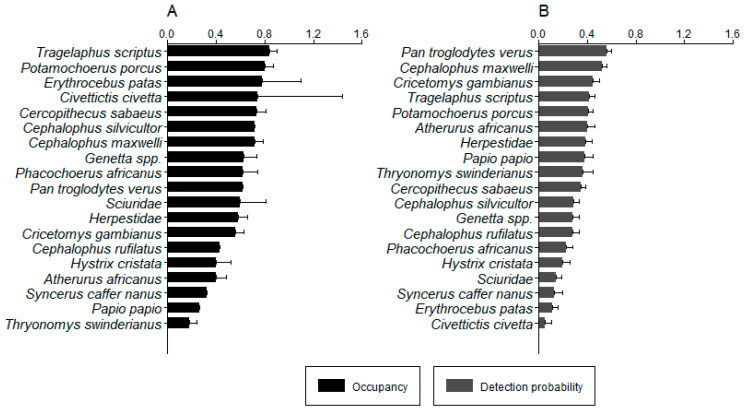
(**A**) Occupancy of the 19 species that recorded at least 10 independent events across the Mafou FPA, UNNP; (**B**) Detection probability of the 19 species that recorded at least 10 independent events across the Mafou FPA, UNNP. Note: Occupancy is the probability that the area is occupied by the species. Detection probability refers to the probability that the species will be detected at least once, when it is present.

Based on our results (Figure 4) the species that emerge as the most widely distributed within the Mafou FPA’s mammal community are *Tragelaphus scriptus*, *Potamochoerus porcus*, *Cercopithecus sabaeus*, *Cephalophus maxwelli*, *Cephalophus silvicultor*, *Pan troglodytes verus*, *Phacochoerus africanus*, the Herpestidae group, *Genetta* spp., *Cephalophus rufilatus*, *Cricetomys gambianus*, *Atherurus africanus*, and *Papio papio.* Camara [5] identified *Cephalophus rufilatus*, *Cephalophus maxwelli*, *Tragelaphus scriptus*, *Phacochoerus africanus*, and *Papio papio* as among the most abundant species in the UNNP. Sociological surveys by Brugière & Magassouba [6] and Duonamou et al. [67] further reinforce this trend, highlighting a paradox: the same species identified as relatively abundant (*Cephalophus rufilatus*, *Cephalophus maxwelli*, *Tragelaphus scriptus*, and *Cephalophus silvicultor*) and *Syncerus caffer nanus* are among the most heavily targeted by poachers.

Our results also indicate that 6 out of 19 species exhibit sensitivity to human disturbances. The occupancy of *Cephalophus maxwelli* and of *Cephalophus silvicultor* declines in areas affected by illegal activities, whereas *Syncerus caffer nanus* shows a positive association with locations characterized by illegal activities. This suggests that *Syncerus caffer nanus* is heavily targeted by armed poachers, a vulnerability previously noted by Diallo [7] and Duonamou et al. [67], while *Cephalophus maxwelli* and *Cephalophus silvicultor* tend to avoid disturbed zones. These findings underscore the need for strengthened surveillance focused specifically on *Syncerus caffer nanus* and its habitat. Meanwhile, *Cephalophus rufilatus*, *Papio papio*, and *Pan troglodytes verus* exhibit higher occupancy farther from Sérékoroba, likely reflecting both the predominance of wooded savannas near the village [68] and the intensity of human pressure there. These results could be somehow spatially biased due to uneven sampling of illegal activities data across the various cells. We recognize that camera performance may vary by type, but the cameras used have the same shutter speeds, depth of field, and other key shared characteristics. This minimizes biases resulting from differences in camera types. In short, unmodelled variations in detectability may have affected the results.

Several threatened species according to the IUCN Red List (V2025-1) remain among the least represented in our detections. Their observation confirms their presence, but does not permit robust conclusions regarding their abundance or occupancy. Brugière et al. [69] identify the UNNP as Guinea’s most important refuge for *Hippopotamus amphibius*, but that earlier estimate around 100 individuals should be revisited in future studies. The under-detection of *Hippopotamus amphibius* in our dataset likely reflects our sampling design, which focused on forest habitats where the species is largely absent [70,71]. Likewise, species such as *Panthera pardus*, *Smutsia gigantea*, *Phataginus tricuspis*, and *Colobus polykomos* remain poorly documented in the UNNP and should receive particular attention in future conservation efforts. The detections of *Smutsia gigantea*, *Phataginus tricuspis*, and *Colobus polykomos*, all semi-arboreal species should be considered a bonus, as their effective monitoring requires specialized arboreal camera-trapping protocols [72,73,74]. Although our study design does not allow robust conclusions about their detection rates or occupancy, the results nonetheless confirm that the UNNP remains an important refuge for these species.

The UNNP emerges as a major reservoir of biodiversity, with species richness comparable to other significant protected areas or biodiversity hotspots in Sub-Saharan Africa such as the Batéké Plateau National Park (BPNP) in Gabon, and the Fazao-Malfakassa National Park (FMNP) in Togo (Table 4). Situated at the transition between the south-eastern edge of the Gabonese rain forest and the north-western limit of the savannah-dominated, BPNP comprises a transitional landscape in which forest galleries along the Mpassa River and its tributaries reach out into the savannah. For its part, Fazao-Malfakassa National Park is the largest national park in Togo and one of the most important protected areas in this country, representative of the semi-deciduous forest-savanna mosaic of the Guinea-Sudanese transition zone in West Africa.

The forests of the UNNP hold considerable ecological value in terms of species richness and RAI, comparable to that of Batéké Plateau National Park and Fazao-Malfakassa National Park, both similar study areas. Across the four sites presented in Table 4, rarefaction curves show a similar tendency toward asymptotic completeness, indicating that each inventory can be considered broadly exhaustive. Although detection rates in the UNNP are relatively lower than those reported for BPNP, they are similar to those of FMNP, underscoring the UNNP’s importance for biodiversity conservation. Moreover, when compared with the Lama Classified Forest (LCF) in Benin, the largest remnant of dense natural forest in the Dahomey Gap home to only seven species of mammals, and with the Luki Biosphere Reserve (LBR) in the Democratic Republic of Congo (14 taxa belonging to 12 families and 5 orders), the UNNP clearly emerges as a protected area of exceptional biological diversity [75,76,77]. Furthermore, these comparisons would be limited due to differences in sampling design, habitat type, sampling effort, camera placement, and season.

**Table 4:** Comparison of species richness and detection rates of some iconic species, resulting from this camera trap survey and similar studies in Gabon and Togo.

Characteristic Elements of the Study	UNNP, Guinea (This Study)	BPNP, Gabon (Hedwig et al. [78])		FMNP, Togo (Assou et al. [79])
		Western Sector	Eastern Sector	
Ecoregion	Guinean forest-savannah mosaic	Western-Congolian forest-savannah mosaic	Western-Congolian forest-savannah mosaic	Guinean montane forests
Sampling effort (camera-days)	4239	2947	2955	9007
Number of cameras	53	20	20	100
CT orientation	Trail	Trail	Trail	Trail
Species richness	30	27	29	32
**Detection rates (per 100 camera-days)**				
*Panthera pardus*	0.166	0.7	0.6	/
*Alcelaphus buselaphus major*	0.142	/	/	0.444
*Nandinia binotata*	0.071	0.1	/	0.022
*Atherurus africanus*	2.595	0.8	4.7	2.443
*Smutsia gigantea*	0.024	0.2	0.4	/
*Tragelaphus scriptus*	5.733	/	0.1	6.106
*Cephalophus silvicultor*	1.911	16.7	11.9	/
*Cephalophus rufilatus*	1.605	/	/	1.066
*Potamochoerus porcus*	4.388	7.2	5.7	0.144
*Syncerus caffer nanus*	0.354	0.8	0.5	/
*Civettictis civetta*	0.378	0.8	0.6	1.41

Note: The ecoregions characterizing each study area are taken from Ridder [80] and Haurez et al. [81]. Ecoregion: A major geographic unit, whether terrestrial or aquatic, that corresponds to natural, physical, and biological characteristics and contains assemblages of natural communities that share the vast majority of their species and ecological dynamics. Sampling effort: number of days each trap was deployed at the relevant site(s).

The mammal assemblage of the Mafou FPA consists of prey species, mesopredators, apex predator, seed-dispersing frugivorous, small carnivores, ecosystem-engineering species, and a megaherbivore. Its structure is similar to that described by Bruce et al. [65], which is dominated by herbivores. The mix of species from different trophic guilds is a good bioindicator for the Mafou FPA.

**Figure 5:**
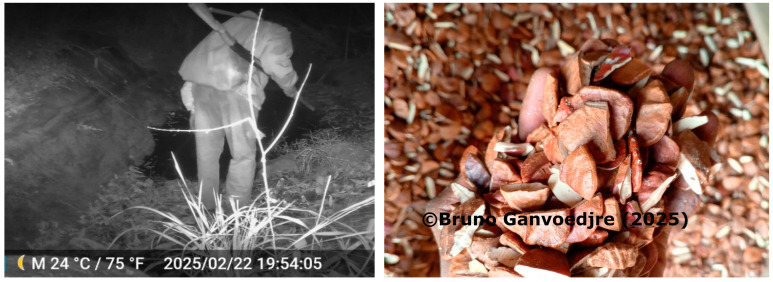
(**Left**)—Armed poacher in the Mafou FPA; (**Right**)—*Detarium microcarpum* fruits, harvested by the riverside women in the Mafou FPA, UNNP, 2025. Note: The human presence index is defined by de Lame et al. [33] such as the number of human detections recorded by the camera traps, divided by the sampling effort (in camera-days), then multiplied by 30 days. This is an indicator of human pressure on the study area, which quantifies the average frequency of human detections by camera traps, standardized over a one-month period.

However, the human presence index at the Mafou FPA scale (0.42) indicates that the mammal community of the Mafou FPA faces considerable anthropogenic pressure (Figure 5). This pressure stems primarily from poaching and the collection of non-timber forest products, particularly the fruits of *Detarium microcarpum*, activities closely linked to the uncontrolled bushfires that affect the area. Both occur during the dry season, when access to the park is easier and vegetation cover is reduced. These activities are illegal and directly or indirectly undermine the biodiversity and ecological integrity of the Upper Niger National Park. In practice, individuals entering the FPA often ignite late-season fires to improve visibility for hunting and fruit collection, inadvertently destroying small wildlife and its habitat. This recurrent issue remains one of the most significant threats facing the UNNP. Beyond the activities of poachers who hunt for profit, many women who collect *Detarium microcarpum* fruit also establish temporary camps with their families inside the Fully Protected Area, relying in part on its wildlife for subsistence. This situation illustrates the multifaceted conservation challenges that persist within the UNNP.

This study provides an important baseline for monitoring and conservation of mammals in the Mafou Fully Protected Area of the Upper Niger National Park. It covered a time scale sufficient to detect the majority of species in the community [23]. By analyzing the mammal assemblage in the area, as well as the distribution of species and their responses to human disturbances, the results of this study in a core area may be useful for conservation strategies for the UNNP. Martin et al. [26] found in Mwanihana forest of Udzungwa Mountains National Park (Tanzania) that neither colonization nor extinction varied with seasons and hence occupancy did not vary, leading them to conclude that seasonal variation in rainfall may have limited effect on occupancy and detectability of resident mammals in Udzungwa rainforests. This supports our analyses, which are based on data from a single season. In addition, a sampling design incorporating both dry and wet seasons, could yield a more robust and ecologically representative assessment of species presence and occupancy. Furthermore, our study design is spatially constrained to the core of the protected area. It is known that terrestrial mammals respond to landscape heterogeneity, particularly to edge effects, gradients in vegetation structure, and varying levels of human disturbance. Future studies could take these aspects into account including also detection covariates such as camera model, habitat type, effort variation as detection predictors and other environmental covariates, to gain a deeper understanding of the factors determining mammal distribution within the landscape.

Finally, the following possible future actions could be implemented for sustainable conservation in the UNNP: (1) strengthening surveillance of this protected area against illegal activities such as armed poaching; (2) framing and structuring the *Detarium microcarpum* value chain; and (3) developing community support initiatives through income-generating activities in the villages bordering the protected area.

## 5. Conclusions

This study provides an initial overview of the mammal community in the Mafou FPA of the Upper Niger National Park, based on camera trap data. It offers important insights into the structure of the mammal assemblage in this community, its distribution, and how this distribution is influenced by human disturbances. As such, it serves as a baseline for monitoring the species comprising this community and is likely to guide future conservation efforts within and around this protected area. Furthermore, the study presents some limitations that are discussed and should be taken into account in future research on mammals in the study area. We primarily recommend that sampling plans account for the two seasons (rainy season and dry season), habitat heterogeneity, the gradient of human impact, and inclusion of covariates that may influence species detection. This, using a multi-season occupancy model, will allow for a more in-depth assessment of the factors that influence the occupancy and detectability of species.

## Figures and Tables

**Table 1:** Species inventoried during the survey through camera traps, detection rates, IUCN status, and mean adult body mass.

Order	Family	Species	Diet	Body Mass (kg)	Number of Detections	Species Detection Rate (RAI)	IUCN Status
Artiodactyla	Bovidae	Western Hartebeest (*Alcelaphus buselaphus*, Blyth, subsp. *major*, 1869) ^1^	Herbivore	120	6	0.142	LC
		Maxwell Duiker (*Cephalophus maxwelli*, Smith, 1827) ^1^	Herbivore	9	297	7.007	LC
		Red-flanked Duiker (*Cephalophus rufilatus*, Gray, 1846) ^1^	Herbivore	14	68	1.605	LC
		Yellow-backed Duiker (*Cephalophus silvicultor*, Afzelius, 1815) ^1^	Herbivore	62.5	81	1.911	NT
		Defassa Waterbuck (*Kobus ellipsiprymnus*, Ogilbyi, subsp. *defassa*, 1833) ^1^	Herbivore	210	6	0.142	NT
		Forest Buffalo (*Syncerus caffer*, Sparrman, subsp. *nanus*, 1779) ^1^	Herbivore	350	15	0.354	NT
		Harnessed Bushbuck (*Tragelaphus scriptus*, Pallas, 1766) ^1^	Herbivore	52	243	5.733	LC
	Suidae	Common Warthog (*Phacochoerus africanus*, Gmelin, 1788) ^1^	Omnivore	77	55	1.298	LC
		Red River Hog (*Potamochoerus porcus*, Linnaeus, 1758) ^1^	Omnivore	80	186	4.388	LC
	Hippopotamidae	Common Hippopotamus (*Hippopotamus amphibius*, Linnaeus, 1758) ^1^	Herbivore	1680	5	0.118	VU
Carnivora	Felidae	Serval (*Leptailurus serval*, Schreber, 1776) ^2^	Carnivore	11.7	1	0.024	LC
		Leopard (*Panthera pardus*, Linnaeus, 1758) ^3^	Carnivore	52.5	7	0.166	VU
	Herpestidae	Herpestidae ^2^	Carnivore	3.2	177	4.176	LC
	Viverridae	African Civet (*Civettictis civetta*, Schreber, 1776) ^2^	Carnivore	12.1	16	0.378	LC
		*Genetta* spp. ^2^	Carnivore	2.1	96	2.265	LC
	Canidae	Side-striped Jackal (*Lupulella adustus*, Sundevall, 1847) ^2^	Carnivore	11.2	1	0.024	LC
	Nandiniidae	African Palm Civet (*Nandinia binotata*, Gray, 1830) ^2^	Carnivore	3.2	3	0.071	LC
Pholidota	Manidae	Tree Pangolin (*Phataginus tricuspis*, Rafinesque, 1821) ^1^	Insectivore	2.55	8	0.189	EN
		Giant Pangolin (*Smutsia gigantea*, Illiger, 1815) ^1^	Insectivore	30	1	0.024	EN
Primates	Cercopithecidae	Campbell’s Monkey (*Cercopithecus campbelli*, Waterhouse, 1838) ^1^	Omnivore	4.3	3	0.071	NT
		Green Monkey (*Cercopithecus sabaeus*, Linnaeus, 1766) ^1^	Omnivore	5.3	169	3.987	LC
		King Colobus (*Colobus polykomos*, Zimmermann, 1780) ^1^	Herbivore	9.84	4	0.095	EN
		Patas Monkey (*Erythrocebus patas*, Schreber, 1774) ^1^	Omnivore	12.6	31	0.732	NT
		Guinea Baboon (*Papio papio*, Desmarest, 1820) ^1^	Omnivore	20.2	53	1.251	NT
	Hominidae	West African Chimpanzee (*Pan troglodytes*, Schwarz, subsp. *verus*, 1934) ^1^	Omnivore	45	566	13.353	CR
Rodentia	Hystricidae	African Brush-tailed Porcupine (*Atherurus africanus*, Gray, 1842) ^1^	Herbivore	2.9	110	2.595	LC
		Crested Porcupine (*Hystrix cristata*, Linnaeus, 1758) ^1^	Herbivore	15	33	0.779	LC
	Nesomyidae	Gambian Pouched Rat (*Cricetomys gambianus*, Waterhouse, 1840) ^1^	Herbivore	0.9	265	6.252	LC
	Sciuridae	Sciuridae ^1^	Herbivore	0.65	27	0.637	LC
	Thryonomyidae	Marsh Cane Rat (*Thryonomys swinderianus*, Temminck, 1827) ^1^	Herbivore	4.2	30	0.708	LC
Not identified	Not identified	Not identified	N/A	N/A	71	1.675	N/A

Note: ^1^ indicates primary consumers/prey. ^2^ indicates secondary consumers/mesopredators. ^3^ refers to a tertiary consumer/apex predator. The conservation status according to the IUCN red list of threatened species (LC: Least Concern; NT: Near Threatened; VU: Vulnerable; EN: Endangered; CR: Critically Endangered; N/A: Not Applicable). Additional species observed in the study area through field observations: Western Kob (*Kobus kob*, Erxleben, subsp. *kob*, 1777) ^1^. Mean body masses were obtained from the literature: Gittleman [48] for carnivores, Wilson et al. [49] for rodents, Demment & Van Soest [50], Steuer et al. [51], and Meireles et al. [52], for artiodactyls and *pholidota*, Hall [53], Ruff [54], Porter [55], Dunbar & Korstjens [56], and Fischer et al. [57] for primates. The number of detections corresponds to the number of independent detections (or independent detection events), defined per species. Detection rate is the mean number of detections per 100 camera-days.

**Table 2:** Occupancy models of species with at least 10 independent detections, and their characteristics.

Species	Model	AIC	Delta AIC	AIC Weight	no.Par.	−2LogLike	Naïve Occupancy	*ψ* (SE)	95% Conf. Interval	*p* (SE)	95% Conf. Interval
*Cephalophus maxwelli*	*ψ*(Hact)*p*(.)	312.76	0.00	0.37	3	306.76	0.71	0.73 *	-	0.52 (±0.04)	0.44–0.6
*Cephalophus rufilatus*	*ψ*(Habdist)*p*(.)	162.87	0.00	0.73	3	156.87	0.35	0.43 *	-	0.28 (±0.05)	0.19–0.4
*Cephalophus silvicultor*	*ψ*(Hact)*p*(.)	256.81	0.00	0.73	3	250.81	0.59	0.72 *	-	0.29 (±0.04)	0.21–0.39
*Syncerus caffer nanus*	*ψ*(Hact)*p*(.)	92.08	0.00	0.43	3	86.08	0.18	0.32 *	-	0.13 (±0.07)	0.05–0.32
*Tragelaphus scriptus*	*ψ*(.)*p*(.)	327.74	0.00	0.6	2	323.74	0.78	0.84 (±0.06)	0.67–0.93	0.42 (±0.04)	0.35–0.5
*Phacochoerus africanus*	*ψ*(.)*p*(.)	206.80	0.00	0.5	2	202.80	0.45	0.62 (±0.12)	0.37–0.82	0.23 (±0.05)	0.14–0.34
*Potamochoerus porcus*	*ψ*(.)*p*(.)	317.91	0.00	0.69	2	313.91	0.75	0.80 (±0.07)	0.63–0.9	0.41 (±0.04)	0.33–0.49
*Herpestidae*	*ψ*(.)*p*(.)	254.20	0.00	0.4	2	250.20	0.53	0.58 (±0.08)	0.42–0.72	0.39 (±0.05)	0.3–0.48
*Civettictis civetta*	*ψ*(.)*p*(.)	88.28	0.01	0.39	2	84.28	0.18	0.74 (±0.67)	0.00–1	0.05 (±0.05)	0.01–0.28
*Genetta* spp.	*ψ*(.)*p*(.)	233.91	0.00	0.71	2	229.91	0.51	0.63 (±0.1)	0.42–0.8	0.28 (±0.05)	0.19–0.38
*Cercopithecus sabaeus*	*ψ*(.)*p*(.)	284.71	0.00	0.73	2	280.71	0.65	0.73 (±0.08)	0.55–0.86	0.35 (±0.04)	0.27–0.44
*Erythrocebus patas*	*ψ*(.)*p*(.)	148.75	0.00	0.46	2	144.75	0.33	0.78 (±0.32)	0.09–0.99	0.11 (±0.05)	0.04–0.24
*Papio papio*	*ψ*(Habdist)*p*(.)	134.91	0.00	0.62	3	128.91	0.24	0.26 *	-	0.38 (±0.07)	0.25–0.52
*Pan troglodytes verus*	*ψ*(Habdist)*p*(.)	278.22	0.00	0.6	3	272.22	0.61	0.62 *	-	0.56 (±0.04)	0.47–0.64
*Atherurus africanus*	*ψ*(.)*p*(.)	198.58	0.00	0.52	2	194.58	0.37	0.4 (±0.08)	0.27–0.56	0.4 (±0.06)	0.29–0.51
*Hystrix cristata*	*ψ*(.)*p*(.)	142.29	0.00	0.39	2	138.29	0.28	0.4 (±0.12)	0.2–0.64	0.2 (±0.06)	0.11–0.35
*Cricetomys gambianus*	*ψ*(.)*p*(.)	258.84	0.00	0.43	2	254.84	0.53	0.56 (±0.07)	0.41–0.7	0.45 (±0.05)	0.36–0.54
*Sciuridae*	*ψ*(.)*p*(.)	147.48	0.00	0.33	2	143.48	0.31	0.6 (±0.21)	0.21–0.9	0.14 (±0.05)	0.06–0.28
*Thryonomys swinderianus*	*ψ*(.)*p*(.)	100.61	0.00	0.47	2	96.61	0.16	0.18 (±0.06)	0.09–0.32	0.36 (±0.09)	0.21–0.54

Note: * indicates a mean occupancy value for species whose occupancy is believed to be influenced by a covariate. *ψ*: occupancy; *p*: detection probability. SE: standard error; AIC: Akaike’s Information Criterion; no.Par.: Number of parameters.

**Table 3:** Untransformed Estimates of coefficients for covariates (Beta’s).

			Coefficients for Covariates (Beta’s)				
Species	Site Covariate	*ψ*.a1 (SE)	95% Conf. Interval		*ψ*.Site Covariate (SE)	95% Conf. Interval	
			Low Value	High Value		Low Value	High Value
*Cephalophus maxwelli*	Hact	0.56 (±0.43)	−0.28	1.4	0.44 (±0.37)	−0.29	1.17
*Cephalophus rufilatus*	Habdist	−5.01 (±1.6)	−8.15	−1.87	0.27 (±0.09)	0.09	0.45
*Cephalophus silvicultor*	Hact	1.68 (±0.77)	0.17	3.19	−0.61 (±0.35)	−1.3	0.076
*Syncerus caffer nanus*	Hact	−1.38 (±0.84)	−3.03	0.27	0.52 (±0.33)	−0.13	1.17
*Papio papio*	Habdist	−3.22 (±1.03)	−5.24	−1.2	0.12 (±0.05)	0.02	0.22
*Pan troglodytes verus*	Habdist	−1.33 (±0.77)	−2.84	0.18	0.11 (±0.05)	0.01	0.21

Note: *ψ*.a1 estimate represent a reference values, and the *ψ*.site covariate estimate represent the slope of the covariates’ influence.

## Data Availability

The data used in this study belong to the Chimpanzee Conservation Center (CCC) and are available from the authors on reasonable request.

## Supplementary Materials

The following supporting information can be downloaded at: https://www.mdpi.com/article/10.3390/ani16142151/s1. The full model-selection results. For each of the 19 species with at least 10 independent detections, the table include all candidate models, AIC, ΔAIC, AIC weight, model likelihood, number of parameters, and −2*LogLike.
