# Supplementary figures and images for: Using Camera Trapping to Assess the Status of the Mammalian Community in the Mafou Fully Protected Area, Upper Niger National Park (Guinea)
Source: Animals (Basel). 2026 Jul 11;16(14):2151. doi: 10.3390/ani16142151 (PMC13404476; doi:10.3390/ani16142151)

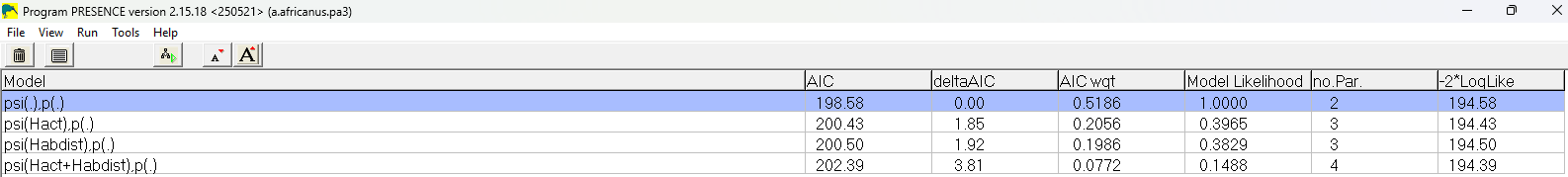

Supplement: Supplementary file 1 [file animals-16-02151-s001.zip › Atherurus africanus.png]

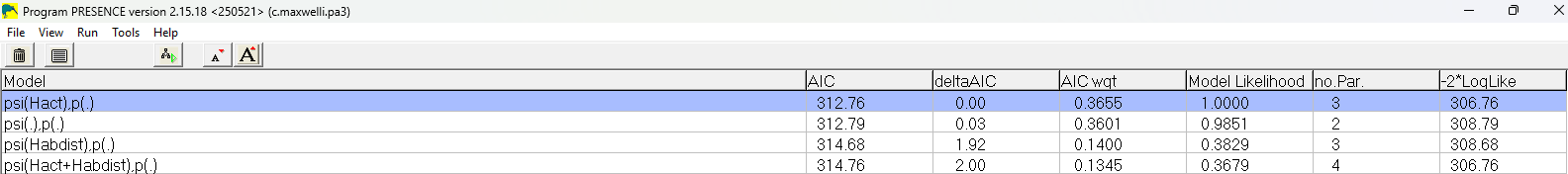

Supplement: Supplementary file 1 [file animals-16-02151-s001.zip › Cephalophus maxwelli.png]

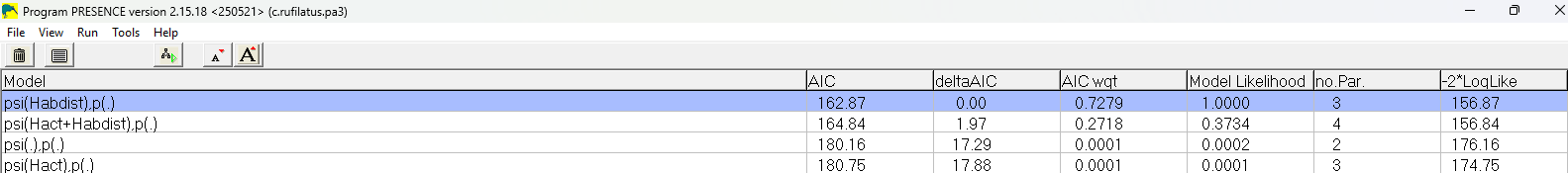

Supplement: Supplementary file 1 [file animals-16-02151-s001.zip › Cephalophus rufilatus.png]

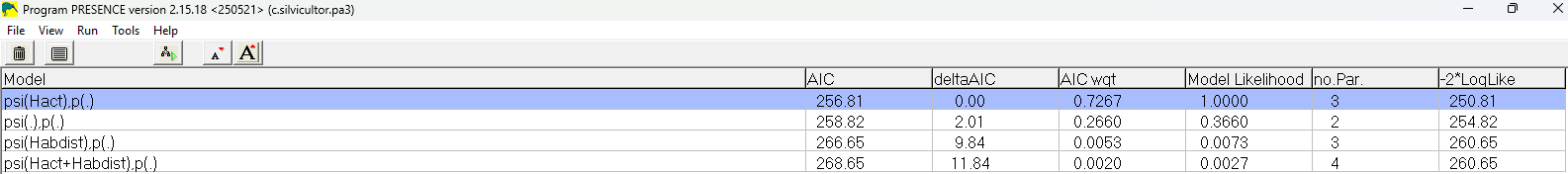

Supplement: Supplementary file 1 [file animals-16-02151-s001.zip › Cephalophus silvicultor.png]

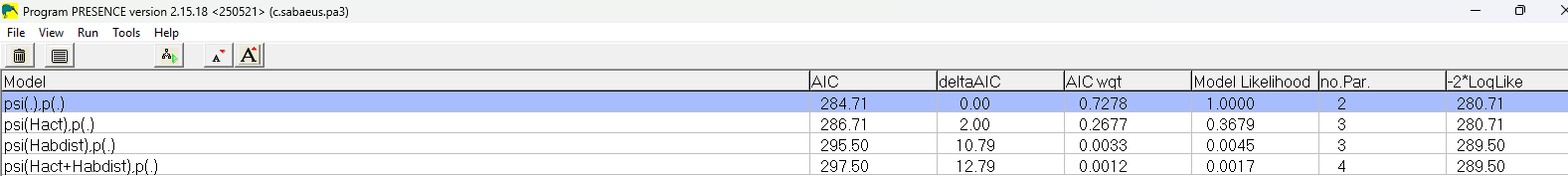

Supplement: Supplementary file 1 [file animals-16-02151-s001.zip › Cercopithecus sabaeus.png]

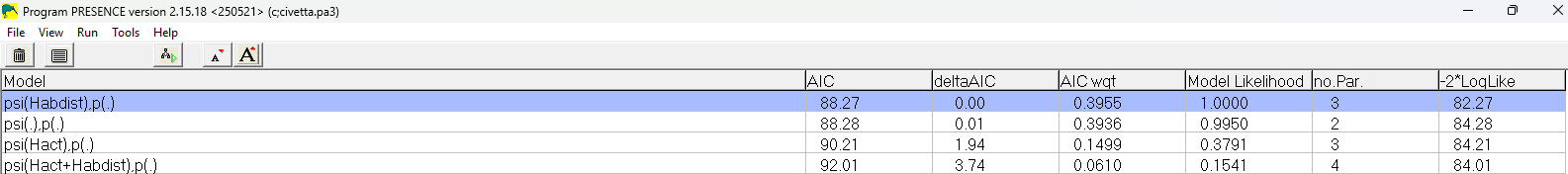

Supplement: Supplementary file 1 [file animals-16-02151-s001.zip › Civettictis civetta.png]

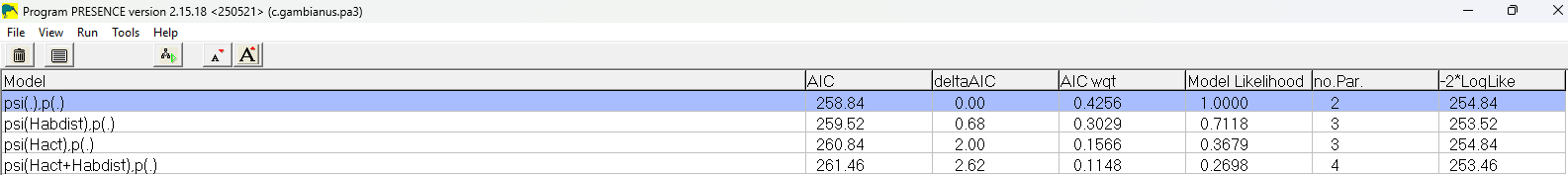

Supplement: Supplementary file 1 [file animals-16-02151-s001.zip › Cricetomys gambianus.png]

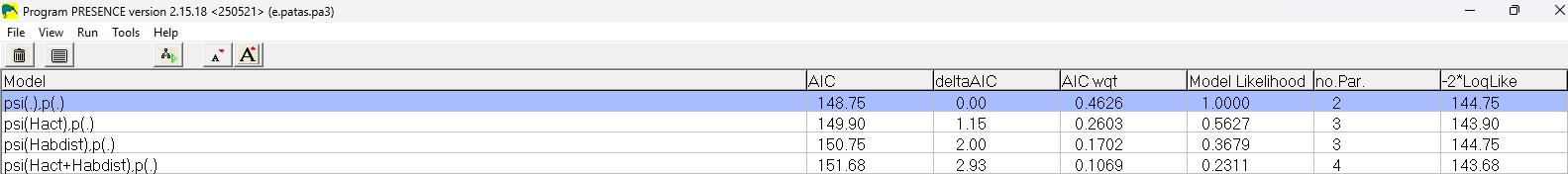

Supplement: Supplementary file 1 [file animals-16-02151-s001.zip › Erythrocebus patas.png]

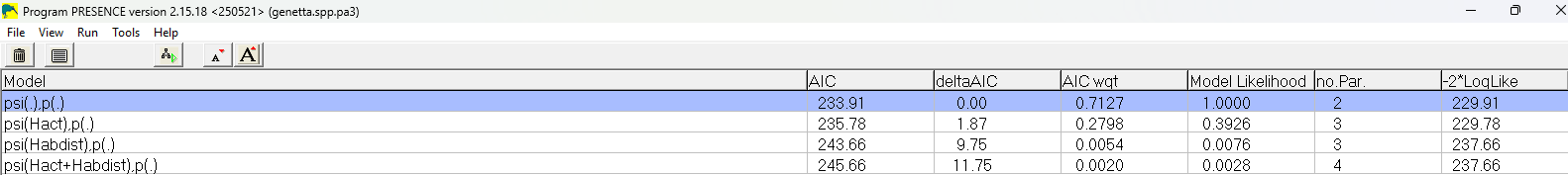

Supplement: Supplementary file 1 [file animals-16-02151-s001.zip › Genetta spp..png]

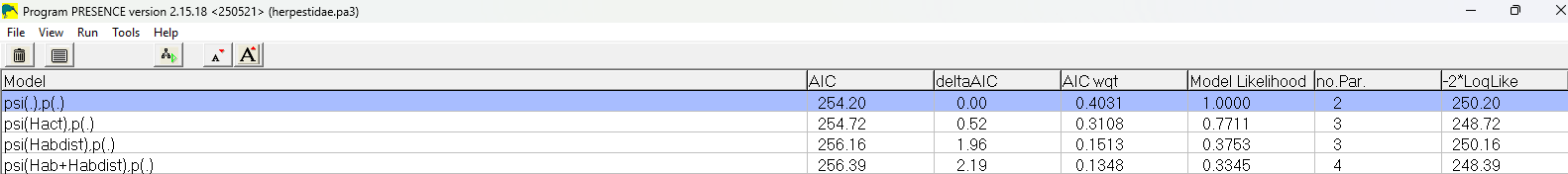

Supplement: Supplementary file 1 [file animals-16-02151-s001.zip › Herpestidae.png]

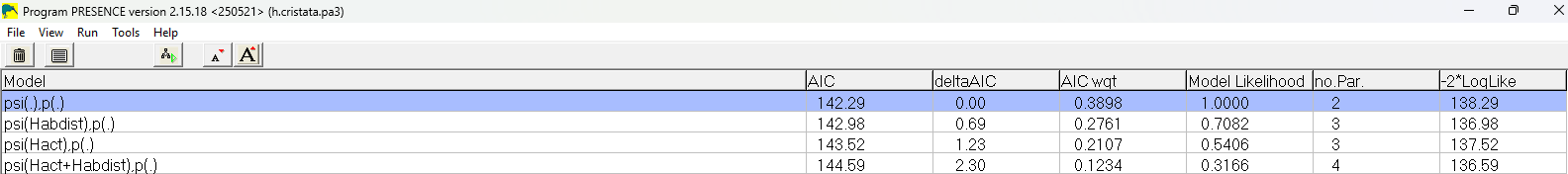

Supplement: Supplementary file 1 [file animals-16-02151-s001.zip › Hystrix cristata.png]

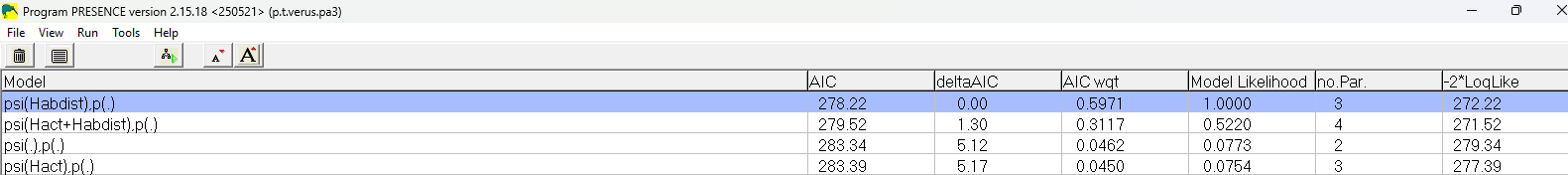

Supplement: Supplementary file 1 [file animals-16-02151-s001.zip › Pan troglodytes verus.png]

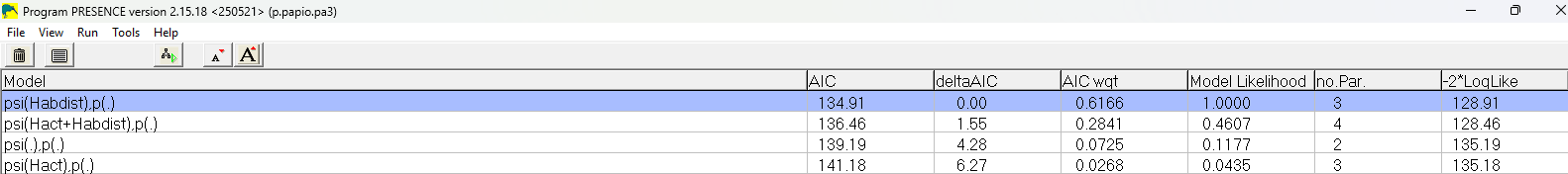

Supplement: Supplementary file 1 [file animals-16-02151-s001.zip › Papio papio.png]

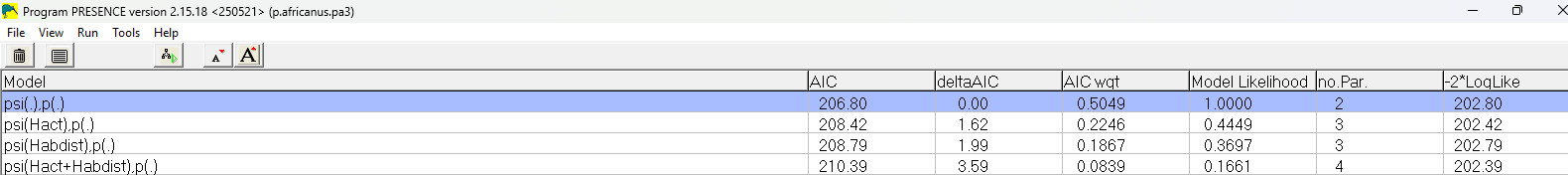

Supplement: Supplementary file 1 [file animals-16-02151-s001.zip › Phacochoerus africanus.png]

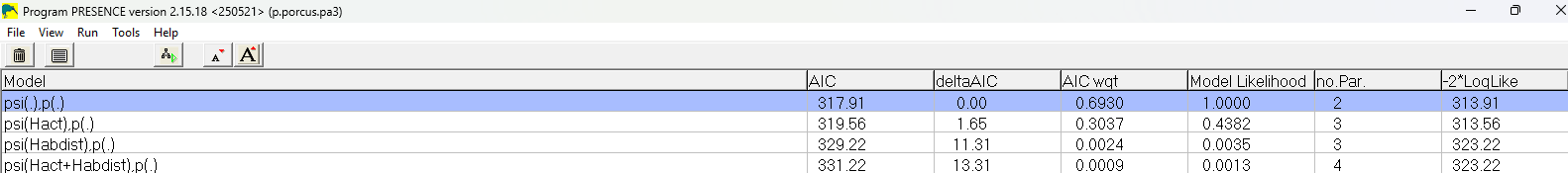

Supplement: Supplementary file 1 [file animals-16-02151-s001.zip › Potamochoerus porcus.png]

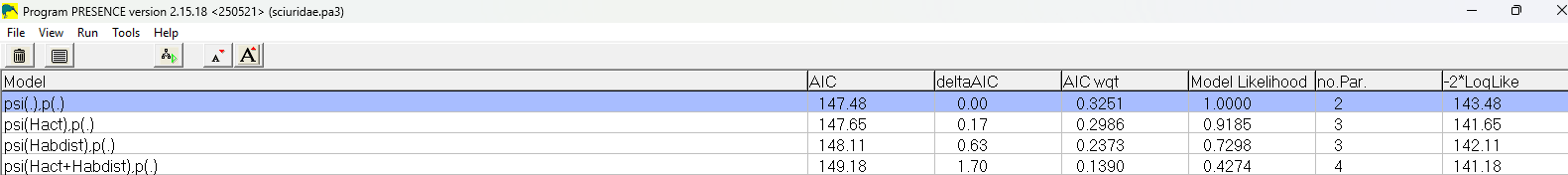

Supplement: Supplementary file 1 [file animals-16-02151-s001.zip › Sciuridae.png]

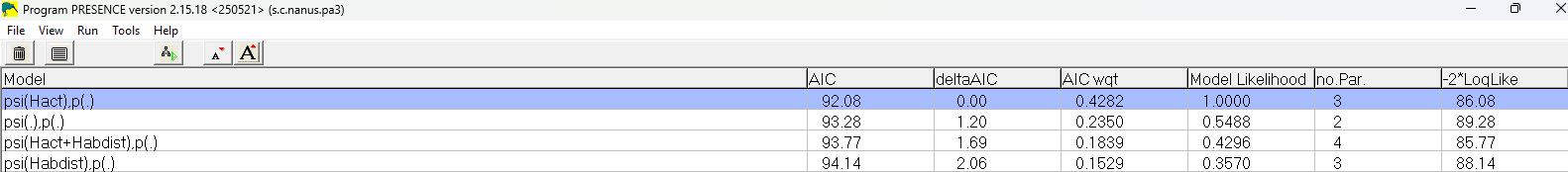

Supplement: Supplementary file 1 [file animals-16-02151-s001.zip › Syncerus caffer nanus.png]

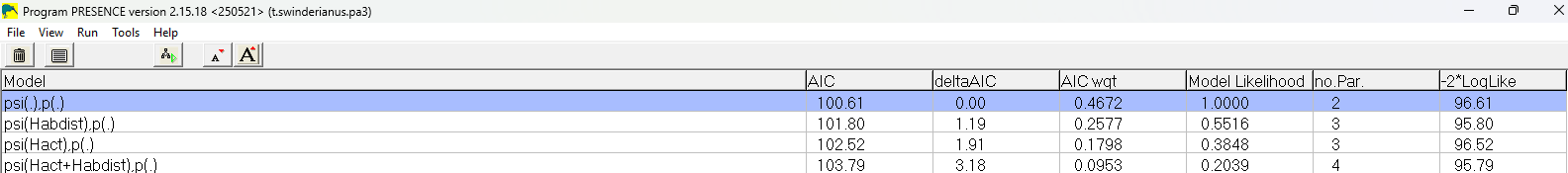

Supplement: Supplementary file 1 [file animals-16-02151-s001.zip › Thryonomys swinderianus.png]

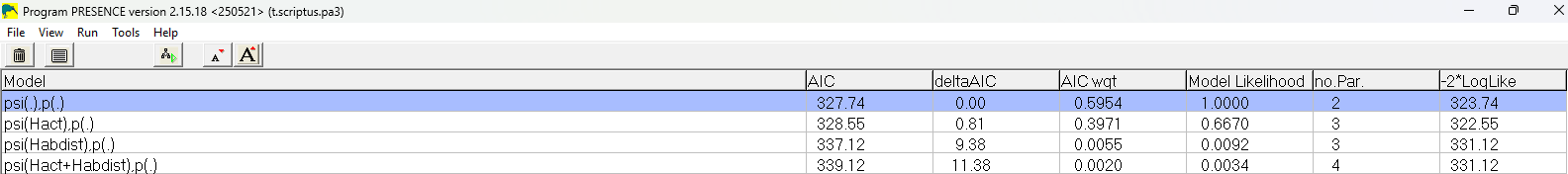

Supplement: Supplementary file 1 [file animals-16-02151-s001.zip › Tragelaphus scriptus.png]
